# “What goes round comes round”

**DOI:** 10.2349/biij.2.1.e1

**Published:** 2006-01-01

**Authors:** BJJ Abdullah

**Affiliations:** Department of Biomedical Imaging (Radiology), Faculty of Medicine, University of Malaya, Kuala Lumpur, Malaysia

Soon after the discovery of x-rays by Roentgen, radioactivity by Becquerel, and radium by the Curies, it was concluded that x-rays could not only be used for diagnostic purposes but also for therapy [1,2]. In the early years of radiation therapy, physicians had little understanding or knowledge of the physical nature and biological effects of radiation, the delivered doses were not certain, the equipment was not just primitive and temperamental but limited in energy output and as such was associated with high failure rates, numerous tumour recurrences and complications. Radiotherapy today has come a long way and has become an essential tool in the treatment of cancer and other diseases along with surgery, chemotherapy, image guided interventions, and targeted radionuclide therapy [3]. In addition, newer biological therapies (immunotherapy, biotherapy, or biological response modifier therapy) are fast becoming part of the widened armamentarium [4]. This special focused issue of ***biij*** will cover some of these topics.

From a common origin based on x-rays, the diagnostic and therapeutic aspects diverged over the last 110 years or so. The early practitioners of radiation therapy and diagnostic radiology began as members of other specialties. Dermatologists and surgeons comprised significant numbers of these early radiation pioneers with even a greater percentage coming from the “field” of electrotherapy. If one reviews some of the early monographs, the titles illustrate the close connection between the two fields of radiation therapy and diagnostic radiology [5,6]. But we seem to have come full circle with the increasing convergence between the therapy and diagnostic aspects with increasingly role of imaging for therapy planning as well as real-time monitoring of therapy, e.g. IMRT [7] and tomotherapy [8]. Convergence is also occurring in the minimally invasive image guided therapy and interventional radiology where there is use of targeted radiotherapy using yttrium embolospheres [9], brachytherapy for the treatment of restenoses following angioplasty [10], radiation synovectomies [11], radiofrequency ablation [12], high intensity focused ultrasound or the use of immunotherapy for the management of lymphoma [3]. Very often these different modalities have to be used in combination to deliver the best effects [13]. The ultimate goal of all the developments and improvements is the effective destruction of only the cancer tissue with little radiation damage to adjacent healthy tissues and to make the treatment easier and shorter for both the patients [14] and the physicians and other healthcare professionals to perform.

The era of molecular medicine with its new insights is having a major effect on the diagnosis, treatment, and prevention of disease and the disciplines of radiology, nuclear medicine, and image-guided therapy and radiation oncology are not exempt. On the contrary, exciting challenges and opportunities in the technologically oriented disciplines of radiology and radiation therapy are leading in new directions other than those mentioned above. The National Cancer Institute (NCI) of USA had outlined a concept that included the three Ds: discovery, development, and delivery where the fields of diagnostic imaging, nuclear medicine, image-guided therapy and radiation therapy can be important contributors to science and health care within this new paradigm [15]. Discovery can be equated with molecular signatures which are the fundamental underpinning, while imaging and therapeutics each comprise the combined components of development plus delivery. At the 2002 RSNA Annual Oration in Radiation Oncology, C. Norman Coleman, MD, who subtitled his presentation as, “Now That Therapy and Diagnosis Have Separated, It's Time to Get Together Again!” reflected his opinion that there is a need for collaboration in research and training among the radiology disciplines with radiation oncologists needing to understand imaging as well as the underlying biologic mechanisms that produce the image findings; the diagnostic radiologists and nuclear medicine physicians in turn need to understand how images are used by radiation oncologists [16]. He went on to add that all trainees need a solid background in basic biology and the new molecular biology techniques. More importantly he stated that the trainees should be sufficiently intrigued and excited by the emerging science to maintain an active interest in clinical and laboratory investigations.

It has been said that when we feel uncomfortable about changes occurring in our environment, we should resist the temptation to withdraw into our comfort zones but rather explore these new possibilities that may herald the dawn of a better era for patients and community. With the increased blurred borders between the various “traditionally” defined disciplines this discomfort is easily appreciated but as has been very succinctly put [17]:

*“Change has a considerable psychological impact on the human mind. To the fearful it is threatening because it means that things may get worse. To the hopeful it is encouraging because things may get better. To the confident it is inspiring because the challenge exists to make things better.”*

*Obviously, then, one's character and frame of mind determine how readily he brings about change and how he reacts to change that is imposed on him.*

*– King Whitney Jr.*

If we take the cue, then the technologically oriented radiological fields – diagnostic imaging, interventional radiology and radiation oncology – have the opportunity to be leaders in these new paradigms (molecular imaging, signatures, and therapy) and we should together aim our efforts in terms of helping with the newest extraordinary opportunity for cancer survivorship [16].
